# Fat deposition in the left ventricle: descriptive and observacional study in autopsy

**DOI:** 10.1186/s12944-017-0475-9

**Published:** 2017-05-02

**Authors:** Ricella Maria Souza da Silva, Roberto José Vieira de Mello

**Affiliations:** 1 Pathological Anatomy Service, Lauro Wanderley University Hospital, Federal University of Paraíba, João Pessoa, Paraíba Brazil; 2 Postgraduate Program in Pathology, Federal University of Pernambuco, Recife, Brazil; 3 Department of Pathological Anatomy, Federal University of Pernambuco, Recife, Brazil

**Keywords:** Adipose tissue, Heart, Necropsy

## Abstract

**Background:**

The human heart contains varying amounts of fat deposits. Cardiac physiological fat occurs predominantly in the right ventricle (RV). The discovery and characterization of adipose tissue along the left ventricle (LV) has been rarely reported. This study aimed to determine the occurrence of fatty deposits in epicardial, pericoronay and myocardial compartments in the LV, and to trace the epidemiological profile and clinical associations with this finding.

**Methods:**

Epidemiological and morphological data and heart samples were collected from corpses submitted to necropsy. Cardiac samples were fixed, embedded in paraffin and subjected to hematoxylin-eosin for microscopic study.

**Results:**

The research was based on 40 samples of cardiac tissue, 21 male cadavers and 19 female ones with mean age of 68.2 years. 52.2% of the subjects had a history of smoking, 20% of them had alcohol consumption and 43.59% showed cardiac cause as a cause of death (acute myocardial infarction – AMI – was the most frequent immediate cause of death). 82.5% of the subjects showed atherosclerotic disease in the ascending aorta (ADAA). The fat deposition in the left ventricule (FDLV) was observed in 95% of cases. Epicardial fat (EF) and pericoronary adipose tissue (PAT) are the most frequent topographies in fat accumulation in the left heart chamber and the EF deposition is associated with myocardial adiposity (MA) (Fisher test [FT] 0.019; odds ratio [OR] 0.097 [95% CI 0.033 to 0.284]; *p* < 0.05). FDLV was associated with alcoholism (FT 0.04, OR 0.161 [95% CI 0.072 to 0.36]; *p* < 0.05); smoking (FT 0.508; OR 0581 [95% CI 0.431 to 0.73]; *p* < 0.05), presence of Frank’s sign (FT 0.502; OR 0.567 [95% CI 0.414 to 0.775]; *p* < 0.05); ADAA (0.774 OR [95% CI 0.6405 to 0.936]; *p* < 0.05); AMI (OR 0.730 [95% CI 0.600 to 0.888]; *p* < 0.05) and macroscopic finding of cardiac hypertrophy (OR 0.700 [95% CI 0.525 to 0.933]; *p* < 0.05). FDLV is related with the thickness of the abdominal fat cushion.

**Conclusions:**

FDLV is common and associated with cardiovascular disease risk factors. Cardiac adiposity cannot be considered a random autopsy finding, requiring diagnostic research and more studies to investigate the clinical implications.

## Background

The human heart contains varying amounts of fat deposits [1]. Physiological cardiac fat can be separated into two compartments: extrapericardic and intrapericardic. Intrapericardial adipose tissue includes EF, PAT and MA [2].

Cardiac physiological fat predominantly occurs in the RV [3, 4] and is present in more than 50% of healthy elderly [5, 6]. The degree of fat in the RV increases with age, and its development is considered part of the aging process [7]. Fat in the pathological myocardium is known to be seen in patients with various heart diseases, such as: healed myocardial infarction [8], right ventricular arrhythmogenic dysplasia [1, 9] and some cardiomyopathies [10, 11].

The finding and characterization of adipose tissue along the LV in healthy individuals or individuals without a clinical history of heart disease has been rarely reported and remains little known [9, 10]. In the last decade, especially due to the rapid development in the noninvasive imaging field, there has been increasing evidence regarding to the presence of fat cells in the myocardium [2].

The present study sought to determine the occurrence and characterization of fat deposits in the different compartments of the LV, tracing the epidemiological profile and clinical associations with this finding, in order to discuss the need for diagnostic and prophylactic investigation of FDLV.

## Methods

### Type and study group

Prospective, cross-sectional, descriptive and observational study. The research was performed with samples of cardiac tissue collected from corpses submitted to necropsy (adults over 18 years of age and of both genders) at the Death Verification Service (DVS) of Recife, an agency linked to the Health Department of the State of Pernambuco and agreed to the Federal University of Pernambuco, followed up for 1 month.

### Collect of data

Data collection was performed using a specific pre-prepared form, using the anatomicopathological cadaveric report and the death certificate made at the time of the necropsy as data sources. Epidemiological and morphological information were collected during the usual necroscopic procedures of death verification (necropsy according to the precepts of the Virchow technique).

The epidemiological data analyzed were: sex; age; pathological background; habits and customs (alcoholism, smoking); immediate cause and basic cause of death.

The morphological data were collected as: absence or presence (unilateral or bilateral) of the diagonal lobular fold; observation of the absence or presence of ADAA; measurement (with centimetric ruler and transparent millimeter) of the thickness of the adipose cushion of the abdominal wall at the height of the umbilical scar and main macroscopic cardiac findings.

### Collect of samples

First, the heart was analyzed macroscopically, observing characteristics related to volume and consistency. The cardiac collection (concomitant to the method of finding the cause of death) followed the following norms: serial cross-sections using a cutting tool along the short axis of both ventricles to the tip of the papillary muscles.

The sections were made parallel to the atrioventricular groove at intervals of 1 to 1.5 cm from the apex of the heart, with the atrioventricular valve apparatus left intact in the remainder of the sample. For the histological analysis, the cardiac transverse slice resulting from the serial cuts, located in the ventricular midline and anatomically with the coronary artery, was selected and fixed in 10% formalin.

**Fig. 1:**
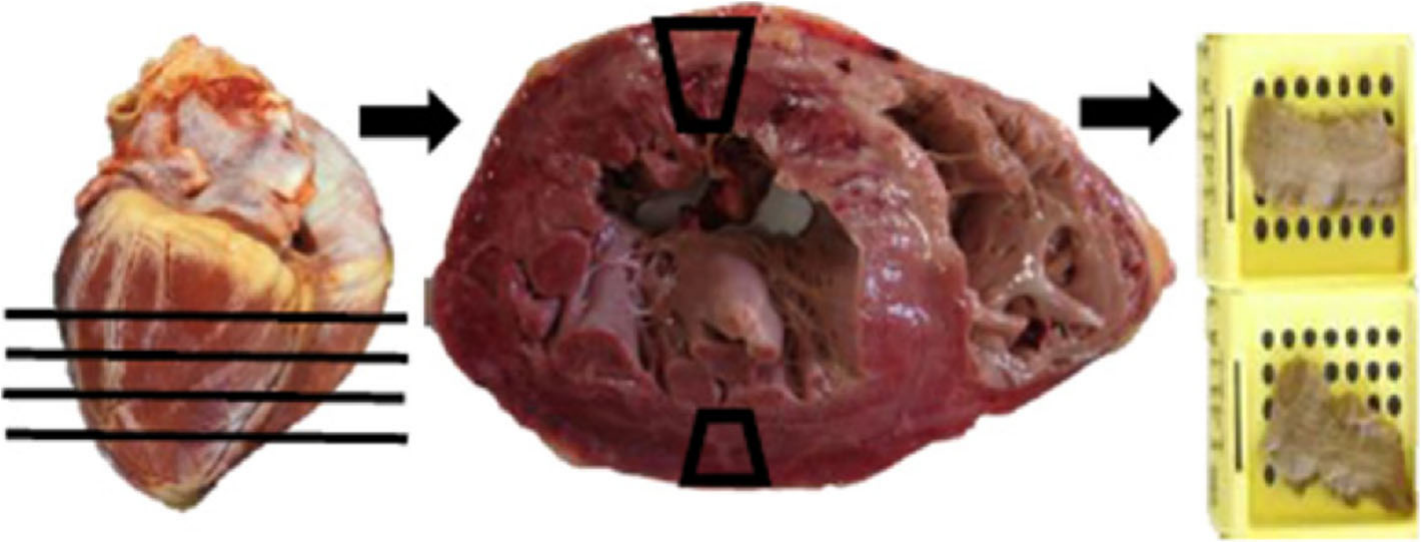
Method of collection of cardiac samples in cadavers submitted to clinical necropsy

From the selected slice, it were collected a sample of the LV anterior wall and a sample of the posterior wall of the LV, placed in one or more histological cassettes (Fig. 1). These samples were submitted to automated histological processing, embedded in paraffin, sectioned, histological sections between 3 and 7 μm thickness, collected on histological slide and stained with hematoxylin-eosin for microscopic study.

### Analysis of the samples

Cardiac microscopic analysis consisted in: a) the identification of the presence of fatty deposition in the anterior and/or posterior wall of the LV; b) the characterization of this deposit in the different anatomical compartments: presence or absence of EF, PAT and MA - intercardiomyocytes; c) measurement with the microscopic ruler (0.5 mm and 10 mm scale) of the greatest thickness of the epicardial adipose deposit for each sample analyzed (microscopic analysis performed on an Olympus BX46 microscope, with Olympus SC30 camera imaging and thickness measurement with microscopic ruler linked to the CellSens Entry software 1.12).

### Ethical aspects

All applicable international, national and/or institutional guidelines were followed. The execution of the present study was authorized by the General Directorate of Health Education of the Health Department of the State of Pernambuco, as well as approved by the Ethics and Research Committee of the Federal University of Pernambuco.

### Statistical analysis

Statistical analysis was performed using SPSS software for Windows version 13.0. The Fisher test was applied for associations between groups of qualitative variables and obtained Risk Chance estimates. Statistical significance was defined as *p* < 0.05.

## Results

### Epidemiological analysis

The research was based on 40 samples of cardiac tissue collected from corpses submitted to necropsy at the DVS of Recife. The study group included 21 male cadavers and 19 female cadavers aged between 35 and 96 years (mean of 68.2 years, median of 70.0 years). The most common pathological antecedent was Arterial Hypertension (35%), followed by Diabetes Mellitus (27.5%) (Table 1). In relation to habits and customs, 52.5% of the individuals had a history of smoking and 20% of alcoholism.

**Table 1:** Pathological history of necropsied corpses in the DVS

	Frequency	Percentage
Arterial hypertension	14	35.0
Cardiopathy	1	2.5
Depression	1	2.5
Diabetes mellitus	11	27.5
Eschar	1	2.5
HIV	1	2.5
Leprosy	1	2.5
Lower member amputation	1	2.5
Muscular dystrophy	1	2.5
No pathological background	5	12.5
Pancreatic neoplasia	1	2.5
Stroke	2	5.0
Total	40	100.0

The most frequent immediate cause of death was AMI (10 cases), followed by sepsis (3 cases). The causes of death were as follows: 43.9%, cardiac diseases; 24%, diseases of the respiratory system; 13%, some infectious or parasitic diseases; 11%, diseases not classified elsewhere; and 5% neoplasms.

### Macroscopic morphological analysis

The diagonal lobular fold - Frank’s sign - was found in 16 of the 40 cadavers analyzed, while 82.5% of the cadavers presented ADAA. A statistically significant association was found between aortic atherosclerotic disease and Frank’s presence (FT 0.029, OR 0.500 [95% CI 0.354–0.707], *p* < 0.05). The thickness of the adipose cushion of the abdominal wall at the height of the umbilical scar varied from 0.5 cm to 9.5 cm (mean of 3.23 cm, median of 2.5 cm). In the examination of the heart, the main macroscopic findings were: AMI, cardiac hypertrophy, pericarditis, cardiomegaly and cardiac dilatation.

### Histological morphological analysis: FDLV

**Fig. 2:**
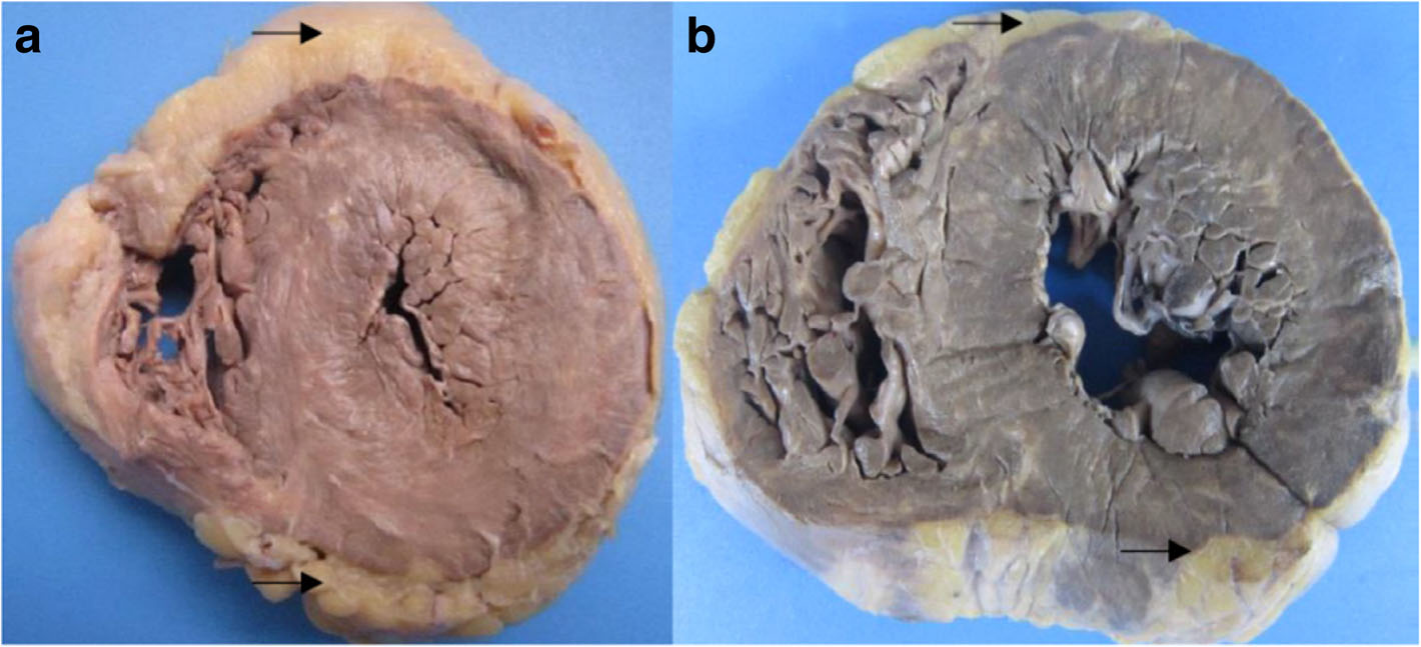
Cross section of the heart with macroscopic view of FDLV (*arrows*). Legend: **a** Case 19; **b** Case 13

**Fig. 3:**
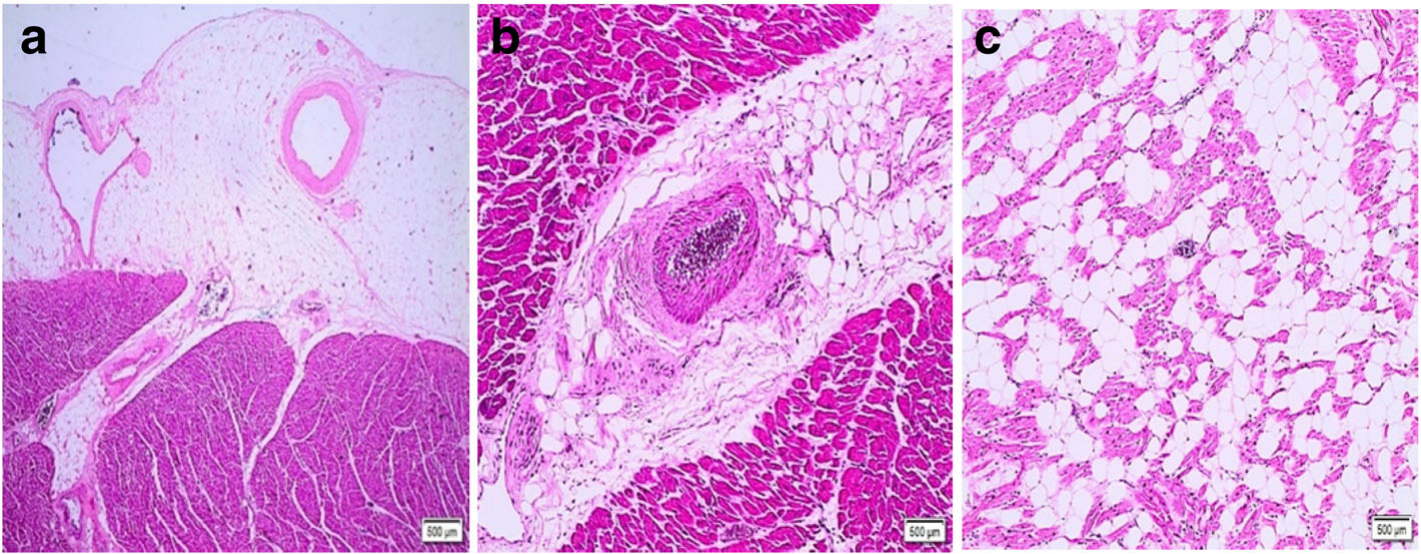
Photomicrographs of cardiac samples showing adipose deposition in anatomical compartments of the LV. Legend: **a** EF (Case 1); **b** PAT (Case 5); **c** MA (Case 7). Hematoxilina-eosina; 4X, 10X

FDLV was observed in 38 of the 40 cases analyzed - 95% of the total (Fig. 2). Considering the different anatomical compartments, the fatty deposit was distributed as follows: 92.5%, EF (Fig. 3); 82.5%, PAT (Fig. 3); and 20% of the cases presented MA (Fig. 3).

**Fig. 4:**
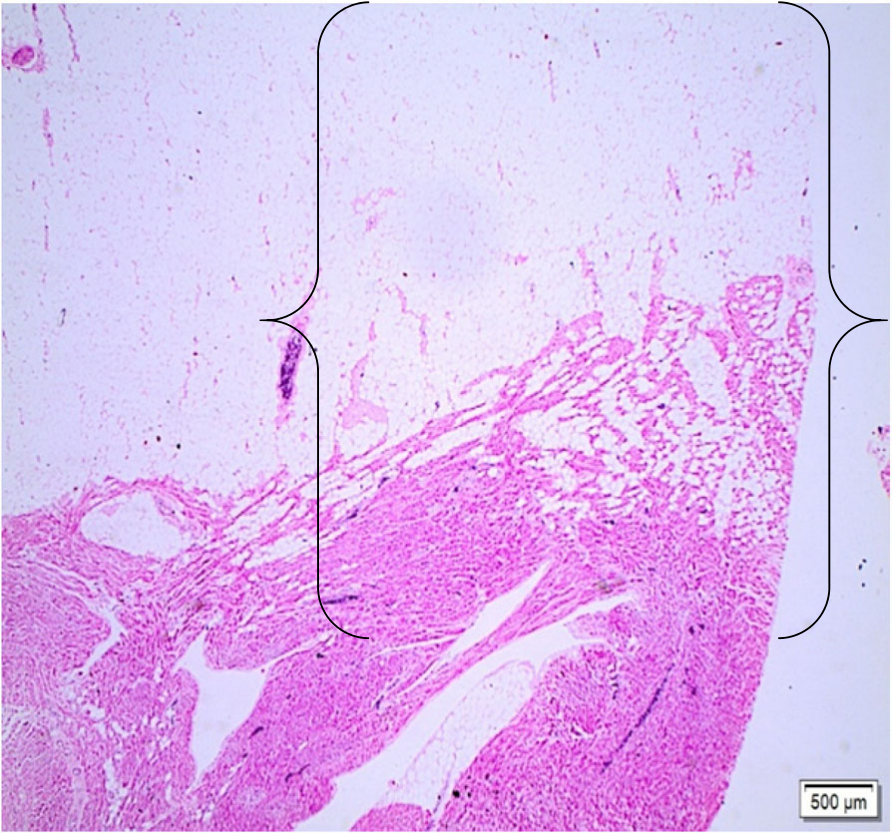
Photomicrograph showing association between EF and AM of the LV (*curly brackets*). Legend: Case 7. Hematoxylin-eosin, 4X

It was observed significance with level of association and chance of risk, between the crossing of variables: a) FDLV and EF (FT 0.006; OR 0.032 [95% CI 0.005–0.22]; *p* < 0.05); b) PAT and FDLV (FT 0.019; OR 0.097; [95% CI 0.033–0.284]; *p* < 0.05); c) AM and EF (FT 0.557, OR 0.758 [95% CI 0.625–0.919], *p* < 0.05) (Fig. 4).

**Fig. 5:**
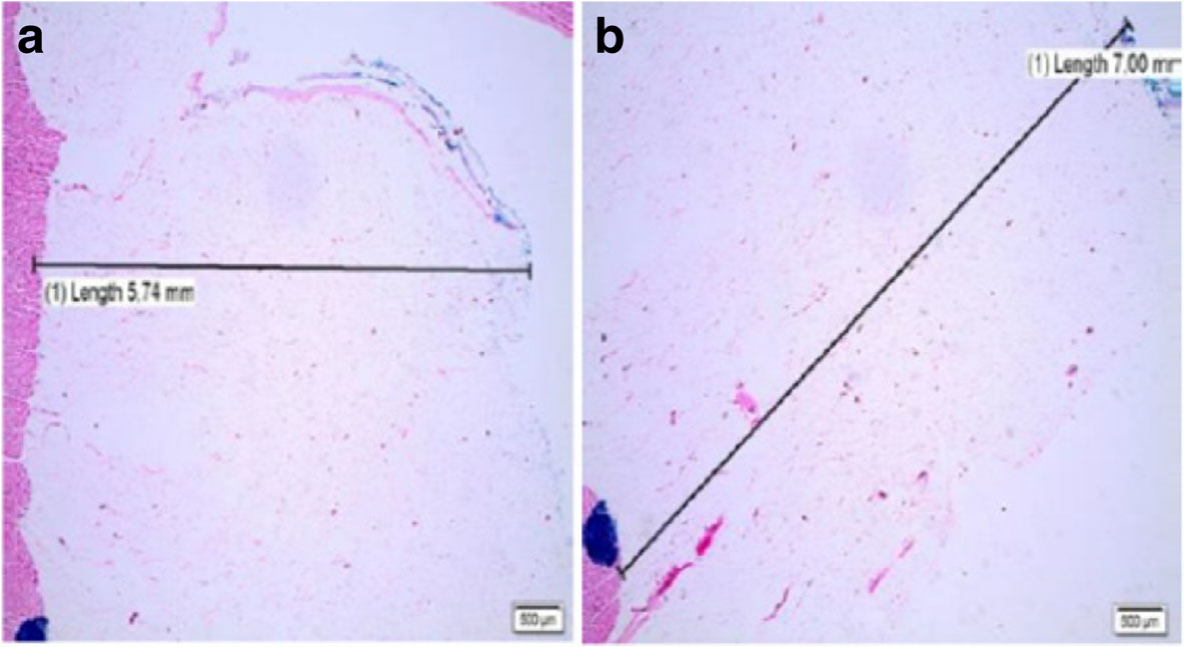
Photomicrographs showing the measurement of the thickness of the EF in the LV. Legend: **a** Case 38: 5.74 mm of fatty epicardial thickness; **b** Case 19: 7 mm of epicardial adipose thickness. Hematoxylin-eosin, 4X

The microscopic measurement of the thickness of EF in the LV ranged from 0.5 mm to 7.0 mm (mean of 2.73 mm, median of 2.4 mm), six samples presented thickness greater than or equal to five (Fig. 5). Statistical correlation was observed between the thickness of the epicardial adiposity and the thickness of the abdominal adipose cushion (r^2^ = 0.5).

### Histological morphological analysis: FDLV x epidemiological and morphological variables

The present study showed an association between the presence of FDLV and the following variables: alcoholism, smoking, presence of Frank’s Sign, ADAA (FDLV present in 93.94% of the 33 cadavers with the disease), immediate cause of death - AMI (FDLV present in all cadavers with AMI) and macroscopic finding of cardiac hypertrophy (Table 2).

**Table 2:** Association between FDLV and epidemiological/morphological variables in cadavers submitted to clinical necropsy

Crossing variables	Fisher statistics	ODDS RATIO (OR)	Inferior limit	Upper limit
FDLV and alcoholism	0.04	0.161	0.072	0.36
FDLV and smoking	0.508	0.581	0.431	0.73
FDLV and Sign of Frank	0.502	0.567	0.414	0.775
FDLV and ADAA	1	0.774	0.640	0.936
FDLV and AMI	1	0.730	0.600	0.888
FDLV and cardiac hypertrophy	1	0.700	0.525	0.933

**Fig. 6:**
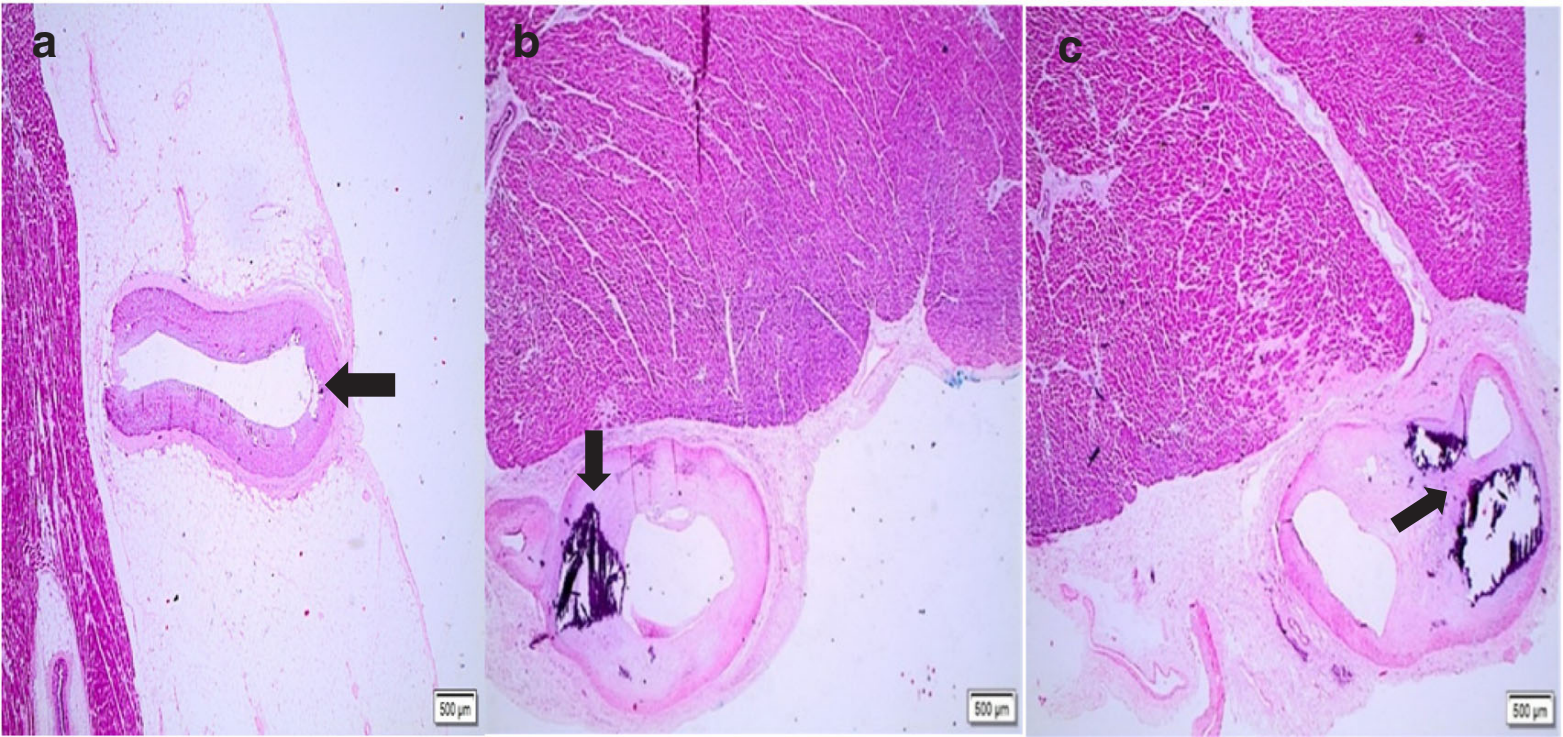
Photomicrographs showing atherosclerotic disease in vessels located inside the EF of the LV: Legend: **a** Case 12 showing atheroma plaque (*black broad arrow*); **b** Case 22 showing calcified atheroma plaque (*black broad arrow*); **c** Case 30 showing atheroma plaque with prominent dystrophic calcification (*broad black arrow*). Hematoxylin-eosin, 4X

In this study, EF was seen in 93.94% of cadavers with ADAA (Fig. 6), and PAT was seen in 31 of the 33 cadavers with ADAA.

## Discussion

In necroscopic studies, cardiac fat is often seen in the RV, with a frequency of up to 85% [5]. Adipose tissue in the left heart chamber, except at the ventricular apex, in healthy individuals has been rarely reported. The necroscopic finding of adipose tissue in the LV in 95% of the cases in the present study reinforces the growing evidence that emerged in the last decade from imaging tests, the presence of fat cells in the heart of healthy people, as well as in people with cardiovascular and non-cardiovascular diseases [2].

Studies indicate that MA is predominantly located in the RV, especially in the anterolateral and apical walls, whereas only a small amount is found in the wall of the LV in its apical portion [7, 12, 13]. In agreement with other studies, no fibrosis or signs of inflammation were observed permeating adipocytes interspersed between the myocardial fibers [7, 12, 14–16]. Bertaso et al. [17] emphasized that the excessive amount of EF tissue may lead to the accumulation of interstitial adipose tissue in the myocardium.

Recent research suggests that EF and epicardial adiposity thickness may be associated with extra-cardiac (aortic and carotid) atherosclerotic lesions [18, 19]. It also suggests that fatty deposition in this compartment may represent an additional and even more direct tool for the stratification of cardiovascular risk, because of its anatomy and functional proximity to the coronary circulation [20]. In addition, perivascular fat can locally accelerate the atherosclerotic process, specifically the coronary [21–24].

According to the current knowledge, abnormal values ​​of EF can be considered as thickness greater than 5 mm [17]. Nelson et al. [25] analyzed the thickness of epicardial adipose tissue through echocardiography and concluded that thicknesses greater than or equal to 5.0 mm could identify an individual with a higher probability of having detectable carotid atherosclerosis (risk factor for cardiovascular disease). All cardiac samples of this study that presented a thickness greater than or equal to five (6 samples) exhibited aortic atherosclerotic disease. Similar to other works, the results indicate that the thickness of epicardial adipose tissue in obese individuals seems to correlate with the abdominal perimeter [26].

The increase in the FDLV may be related to changes in the lifestyle of the population, related to eating habits and sedentary lifestyle, which resulted in overweight and obesity, with consequent adipose tissue deposition in the organic systems.

The results found between FDLV and epidemiological variables reinforce the correlation of cardiac adiposity with markers of cardiovascular disease risk [9, 27], once the increase in cardiovascular risk is associated with the consumption of elevated alcohol levels [28–31], the presence of Frank’s Sign [32, 33] and smoking [34–36]. Recent studies suggest that smoking may have a direct association with fatty cardiac deposition [37, 38].

All cadavers that had AMI as an immediate cause of death presented FDLV. The studies mention a high incidence (68–84%) between the presence of FDLV and AMI, based on necropsy and imaging results [39, 40]. In patients with ischemic heart disease, the presence of FDLV was observed in up to 6% of computed tomography scans [40]. The association measure identified a significant risk chance for these variables.

The association found in this study indicates that the FDLV constitutes a direct risk of cardiac hypertrophy. Kaminaga and others [41] concluded that Hypertrophic cardiomyopathy may be associated with the presence of myocardial fat in the LV with a thickened wall. It has recently been inferred that the accumulation of cardiac fat can generate mechanical overload, resulting in a remodeling of the cardiac mass, alteration of the vascular resistance and the ejection fraction [42], with consequent reduction of the LV performance, eccentric modifications of left ventricular chamber, systolic changes and increased tension in the LV wall [43, 44]. All these anatomical and functional repercussions can be seen from the installation and progression of cardiac hypertrophy.

Likewise, the fatty deposition in the left ventricle was consistently associated with aortic atherosclerosis, being present in 93.94% of the 33 cadavers carrying the disease. The results corroborate with other studies that reported a potential association between the variables, either independently or indirectly [45–48].

These results show that the FDLV may represent an additional tool for the stratification of cardiovascular risk.

## Conclusion

The results obtained in this work demonstrated that FDLV is frequent, with EF and PAT being the most frequent topographies for fatty accumulation in the left heart chamber. In addition, EF is associated with MA. Furthermore, FDLV is associated with heart diseases such as cardiac hypertrophy and AMI and correlates with the thickness of the abdominal adipose cushion. We believe that cardiac FDLV may represent an additional tool for the stratification of cardiovascular risk and cannot be considered a random necroscopic finding, requiring diagnostic investigation and ascertainment of the clinical implications.

## Abbreviations

ADAA: Atherosclerotic disease in the ascending aorta
AMI: Acute myocardial infarction
EF: Epicardial fat
FDLV: Fat deposition in the left ventricule
LV: Left ventricle
MA: Myocardial adiposity
PAT: Pericoronary adipose tissue
RV: Right ventricle
